# Supplementary material for: Heterogeneous thermal tolerance of dominant Andean montane tree species
Source: Commun Earth Environ. 2025 Feb 17;6(1):117. doi: 10.1038/s43247-025-02083-w (PMC11832418; doi:10.1038/s43247-025-02083-w)
Supplement: Supplementary file 2 — Supplementary Material [file 43247_2025_2083_MOESM2_ESM.pdf]

Supplementary material

Heterogeneous thermal tolerance of dominant Andean montane tree species

Zorayda Restrepo[1,2,3*], Sebastián González-Caro[4], Iain P. Hartley[4], Juan Camilo Villegas[1,3], Patrick Meir[5,6], Adriana Sanchez[7], Daniel Ruiz[8] and Lina M. Mercado[4,9*].

[1]Grupo GiGA, Escuela Ambiental; Facultad de ingeniería, Universidad de Antioquia, Medellín, Colombia; [2]Grupo de Servicios ecosistémicos y Cambio Climático, Corporación COL-TREE, Medellín, Colombia; [3]Grupo en Ecología Aplicada, Escuela Ambiental, Facultad de ingeniería, Universidad de Antioquia, Medellín, Colombia; [4]Geography, Faculty of Environment, Science and Economy, University of Exeter, Exeter, United Kingdom; [5]School of Geosciences, University of Edinburgh, Edinburgh, United Kingdom; [6]Research School of Biology, Australian National University, Canberra, Australia; [7]Programa de Biología, Facultad de Ciencias Naturales, Universidad del Rosario, Bogotá, D.C., Colombia; [8]Sistema de Alertas Tempranas de Medellín y el Valle de Aburrá, SIATA, Medellín, Colombia; [9]UK Centre for Ecology & Hydrology, Wallingford, United Kingdom.

*Corresponding authors: corporacioncoltree@gmail.com, l.mercado@exeter.ac.uk

Supplementary Notes 1 Criteria used for selecting fertilizers

Supplementary Table 1 Summary of species level size average at each experimental site measured in January 2022 three years after planting: stem diameter (D), total stem height (H), and maximum canopy diameter (C).

Supplementary Table 2 Soil macronutrients and pH (soil characteristics) of native forest soils, soils used for planting, recommended values in the literature and soils used on fertilised trees.

Supplementary Table 3 Nutrient quantities and frequency added per tree during planting and over experimental period on fertilised trees.

Supplementary Table 4 Concentration of macronutrients and micronutrients in applied fertilisers.

Supplementary Table 5 Composition of Organic Humic Acid at 12.7%.

Supplementary Table 6 Nutrient and texture analysis from forest soils where seeds were originally collected from nearby the 14 °C MAT experimental site. C=Clay, Si=Silt, Sn=Sand, OM is organic matter, and CEC is effective cation exchange capacity.

Supplementary Table 7 Nutrient and texture and analysis from soils where trees were planted. C=Clay, Si=Silt, Sn=Sand, OM is organic matter, and CEC is effective cation exchange capacity.

Supplementary Figure 1 Location of the study area and experimental sites in the tropical Andes.

Supplementary Figure 2 Growth rate trends over time.

Supplementary Figure 3 Relationship of scaled growth rate (SGR) and species thermal displacement from thermal optimum (TDI).

Supplementary Figure 4 Density plot of temperature and elevation across the Colombian Andes.

Supplementary Notes 1 Criteria used for selecting fertilisers

Tree species differ in nutrient requirements and nutrient use strategies[1]. As a result, nutrient limitations can vary across species within the same site, and the impact of nutrients on tree growth can be species-specific[2–4]. To determine the nutrient quantity to apply per tree to fertilised trees during planting and throughout the experimental period, nutrient analysis on the original forest soils where the seeds were collected from (nearby the 14°C MAT experimental site) and on the soils used for planting were conducted. Overall, forest soils are high in N and K with low values of P, Ca, Mg and pH (Supplementary Table 1) compared to the reference values for intermediate fertility soils. Soil for planting had relatively high levels of Nitrogen (N) and Calcium (Ca), but low levels of Phosphorus (P), Potassium (K), Magnesium (Mg), and was moderately acidic in pH (Supplementary Table 2) compared to the reference intermediate fertility soils (Supplementary Table 3).

The fertilisation strategy aimed to remove nutrient deficiencies on fertilised trees using as reference values from intermediate fertility soils[5-8]. Therefore, we aimed to increase P, K, Mg and pH while maintaining N and Ca (Supplementary Tables 1-3). Very small but frequent additions were applied to maintain moderate nutrient input (Supplementary Tables 3-6), as neotropical montane rainforests and tropical tree species typically respond rapidly to moderate nutrient additions, which influences productivity and growth[1,5].

Estimation of amounts of fertiliser used as reported in Supplementary Table 3 We planted 360 trees, 2.5 m distance apart, in 2400 m^2. Using the same planting distance, this is equivalent to 1500 trees per ha. To evaluate nutrient addition, we express all macronutrients in Kg ha^{-1} within the top 30cm of soil using an apparent soil density of 1 Ton m^{-3}, for a total soil mass of 3,000,000 kg ha^{-1}(Supplementary Table 3). N values were derived from the organic matter content, where N represents 5%, but only 1% is available to plants. P was directly converted from ppm to kg based on the soil mass per hectare. Nutrients reported in milli equivalents (meq) of the nutrient in question in 100g of soil require conversion from meq to grams. For this, each element is divided by its molecular weight and valency, then converted into grams in 0.1 kg (since the soil test report is in meq in100g of soil), and finally adjusted for the soil mass per hectare.

Supplementary Table 1 Summary of species level size average at each experimental site measured in January 2022 three years after planting: stem diameter (D), total stem height (H), and maximum canopy diameter (C).

Species	Planting size		14°C MAT Site			22 °C MAT Site			26°C MAT Site		
	D (mm)	H (cm)	D (mm)	C (cm)	H (cm)	D (mm)	C (m)	H (cm)	D (mm)	C (m)	H (cm)
Inga ingoides	6.17±4.91	57.1±23	30.27±14.64	89.8±62.1	95.2±41	73.8±20.95	213.5±72.9	147.1±334	195.5±90.61	451.7±276.2	425.6±165
Inga marginata	8.18±4.57	90.2±21	34.82±11.3	91.4±41.7	105.6±43	74.13±20.37	132.5±42.4	190.4±437	134.43±45.97	215.3±102.5	296.7±79
Inga spectabilis	6.3±3.65	38.0±18	17.66±7.72	17.8±18.4	48.5±26	56.76±24.56	58.3±36.4	132.8±744	122.26±41.02	186.8±116.2	392.5±153
Inga densiflora	5.84±5.25	50.6±33	30.26±15.66	52.4±43.6	90.5±45	115.55±28.67	212.2±75	310.2±875	46.45±26.51	212.8±276	409±263
Andesanthus lepidotus	5.39±4.97	38.8±32	80.71±41.76	97±64.3	165.2±60	58.8±20.93	10.3±27.2	113.4±80			
Guatteria lehmannii	3.27±3.57	34±17	21.81±12.8	27±20.4	46.8±29	26.87±14.51	9.7±15.9	66.1±261			
Ilex laurina	5.17±4.01	51.6±28	36.05±11.75	45.9±28.5	117±41	24.32±17.13	43.1±24.8	102.1±274			
Quercus humboldtii	5.81±2.77	48.6±16	38.18±11.2	96.1±34.1	155.5±41	20.56±11.54	46±22.7	68.5±334			
Miconia theizans	5.33±4.10	61.7±37	68.52±20.8	87.6±50.1	139.1±40	20.51±25.75	30.2±43.6	115±292			
Clusia multiflora	5.4±5.01	32.8±21	37.04±11.17	56.9±50.2	86.7±28	17.78±19.01	13.6±19.6	62±252			
Clethra fagifolia	3.14±2.85	100±15	36.45±15.87	35.2±19.5	96.8±45	14.68±11.98	11.3±13.3	60.3±237			
Hieronyma antioquensis	3±1.36	48.5±13	29.1±8.61	32.4±16.6	89.3±31	11.85±13.69	9.6±13.3	106.2±223			
Weinmannia pubescens	3.99±3.60	48.5±26	33.02±14.18	71.1±32.9	119.1±53	7.34±10.09	15.1±24.2	69.2±438			
Clusia ducu	3.84±3.44	34.2±15	24.11±14.19	28.4±24.6	60.9±24						
Chrysochlamys colombiana	4.52±3.33	36.5±17	22.42±11.24	23.3±49.4	52.1±24						

Supplementary Table 2 Soil macronutrients and pH (soil characteristics) of native forest soils, soils used for planting, recommended values in the literature[5-8] and soils used on fertilised trees. meq stands for milli equivalents. The combined amount of Ca and Mg used to increase soil pH in planting soil is given, and it comes in a single product.

Soil characteristic	Recommended values	Soil for planting	Native forest soil (kg ha^{-1})	Recommended values (kg ha^{-1})	Soil for planting (kg ha^{-1})	Nutrient applied (g per tree)	Nutrient applied (kg $ha^{-1}year^{-1}$)
Nitrogen (N)	<60 ppm	9.70%	403.5	180	146	97	109.5
Phosphorus (P)	<20 ppm	6.85 ppm	28.5	60	20.55	13.7	36.36
Potassium (K)	<150 ppm	0.46 meq in 100g of soil	678	450	276	184	72.93
Calcium (Ca)	<60 ppm	1.15 meq in100g of soil	90	450	690	460	1008
Magnesium (Mg)	<60 ppm	0.35 meq in 100g of soil	330	450	210	140	657
pH	6-6.5	5.65	4.95	6-6.5	5.65		
Amount of Ca and Mg to increase soil pH							1300

Supplementary Table 3 Nutrient quantities and frequency added per tree during planting and over experimental period on fertilised trees.

Nutrient addition per tree	Planting stage	Monthly	Quarterly
Macronutrients (NPK) and micronutrients Ca, MG, S, Br, Co, Cu, Fe, Mn, Mo, Zn and SiO2) Product composition in Supplementary Table 4	70 g		70 g
Organic Humic Acid 12.7%, Product composition in Table S5	10 cm^3	10 cm^3	
Mycorrhizae	50 g		
Phosphoric rock composed of 30% P_2O_5, 43% CaO and 22% SiO_2.	200 g		
$CaMg(CO_3)_2$	500g		500g

Supplementary Table 4 Concentration of macronutrients and micronutrients in applied fertilisers.

Nutrient	Concentration (%)
Total Nitrogen (N)	12.0
Amoniacal Nitrogen (N)	2.0
Ureic Nitrogen (N)	10.0
P_2O_5	6.0
K_2O	27.0
CaO	5.0
MgO	5.0
S	2.5
B	0.3
Co	0.004
Cu	0.13
Fe	0.05
Mn	0.32
Mo	0.005
Zn	0.3
SiO_2	2.0

Supplementary Table 5 Composition of Organic Humic Acid at 12.7%.

Compound	Amount in g/L
C from the humid extract	127.00
C in humid acid	79.90
C in fluvic acids	47.50
Solids insoluble in water	20.10
K20	19.00 g/L
Na	9.80 g/L

Supplementary Table 6 Nutrient and texture analysis from forest soils where seeds were originally collected near the 14 °C MAT experimental site. C=Clay, Si=Silt, Sn=Sand, OM is organic matter, and CEC is effective cation exchange capacity.

Soil depth used for sampling	pH	OM	P	Al	Ca	Mg	K	CEC	Texture (%)			N
		(%)	(ppm)	meq/100 g de soil					C	Si	Sn	%
0-10 cm	5,0	26,9	10	0,5	0,7	0,7	2,01	3,91	86	12	2	1,40
10-30cm	4,9	25,7	9	2,5	0,3	0,4	0,25	3,45	82	16	2	1,32
30-50cm	5,5	16,8	6	0	0,1	0,1	0,11	0,31	86	12	2	0,87
50-100cm	5,9	10,6	3	0	0,1	0,1	0,09	0,29	86	12	2	0,67

Supplementary Table 7 Nutrient and texture and analysis from soils where trees were planted. C=Clay, Si=Silt, Sn=Sand, OM is organic matter, and CEC is effective cation exchange capacity.

Soil depth used for sampling	pH	OM	P	Al	Ca	Mg	K	CEC	Texture (%)			N
		(%)	(ppm)	meq/100 g de soil					C	Si	Sn	(%)
0-10cm	5.4	10.6	9	0.5	1.5	0.6	0.4	2.95	82	16	2	0.62
10-30cm	5.9	8.8	4.7	0	0.8	0.1	0.1	1.01	72	22	6	0.53
30-50cm	6	9.1	6.9	0	0.9	0.1	0.1	1.05	66	28	6	0.53
50-100cm	5.9	4.6	2.9	0	0.4	0.1	0.1	0.63	82	18	0	0.31

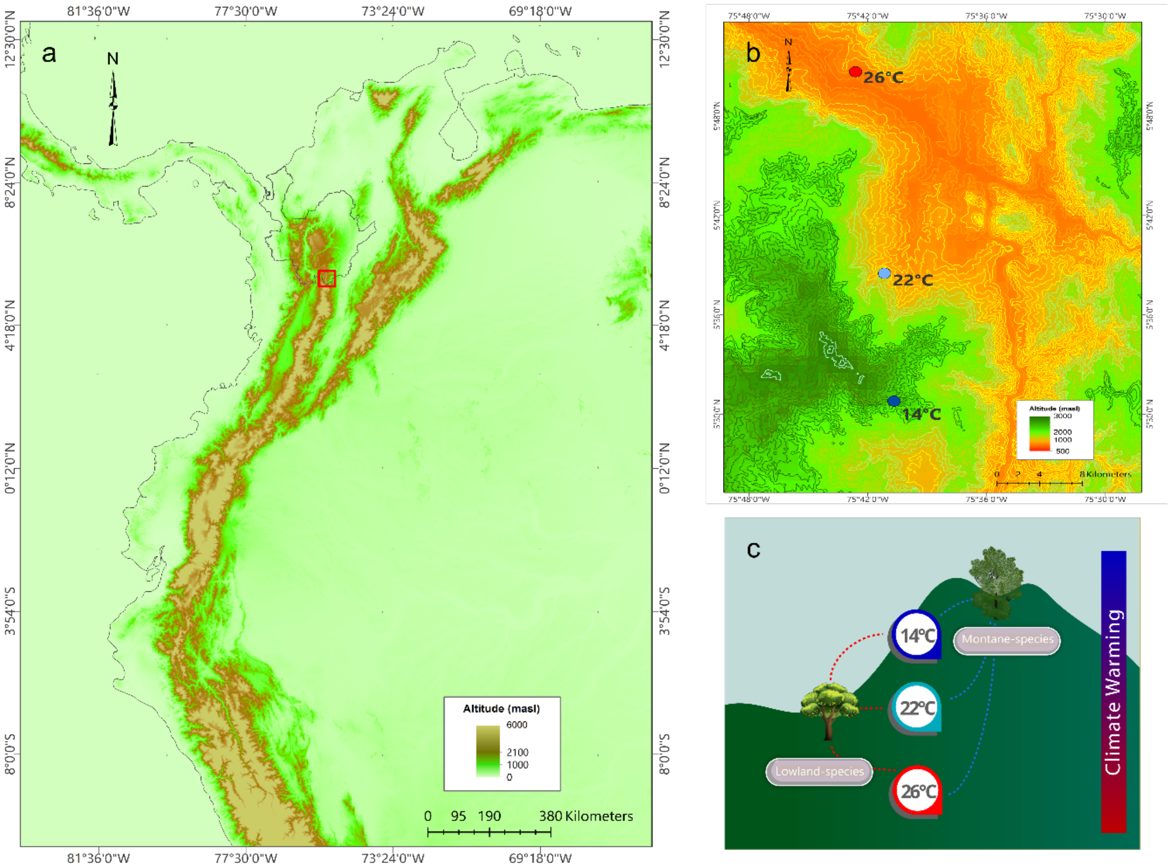

Supplementary Figure 1 | Location of the study area and experimental sites in the tropical Andes. Experimental sites are located in the Antioquia department of Colombia, red grid in panel (a), within a ~2000m elevation gradient (b) with mean annual temperature, altitude, mean annual precipitation and geographical coordinates as follows: 14°C experimental site: 14°C MAT, 2516 masl, 2774 mm year^{-1}, latitude: 5.513277 N, longitude: -75.678311 W, 22°C experimental site: 22°C MAT, 1357 masl, 2045 mm year^{-1}, latitude: 5.641678 N, longitude: -75.685954 W and 26°C experimental site: 26°C MAT, 736 masl, 2298 mm year^{-1}, latitude: 5.844561 N, longitude: -75.710442 W. Experimental setup (c): lowland species with T_{opt} of their thermal distribution closest to 22°C (2 species) and closest to 26°C (2 species) and 11 montane species with T_{opt} closest to 14°C were planted in all experimental sites. Altitudinal distributions in panel (b) were estimated with a digital elevation model.

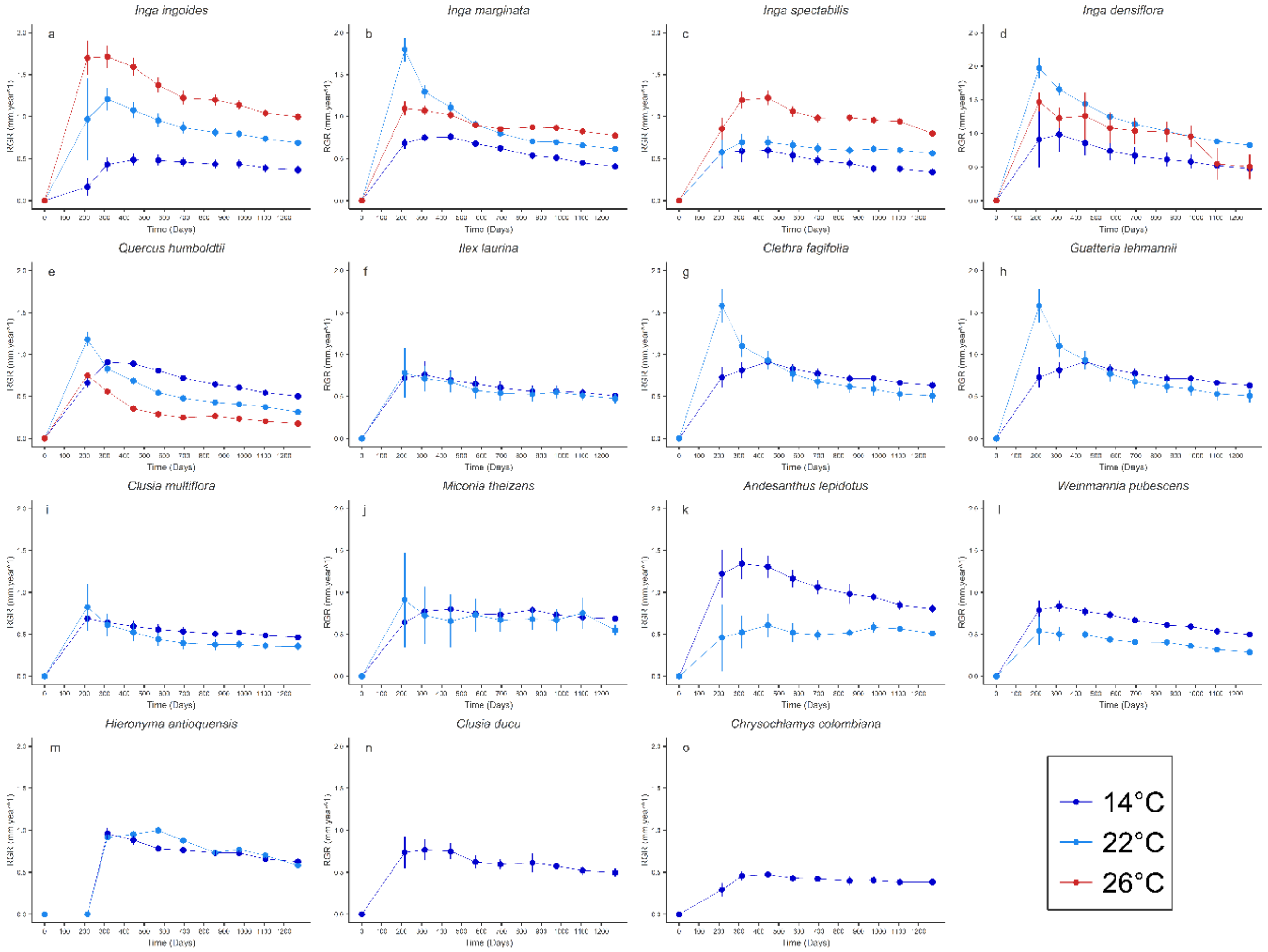

Supplementary Figure 2 | Growth rate trends over time. RGR indicates the response to experimental site MAT (14, 22, and 26°C) at each census (X-axis in days). Error bars correspond to one standard deviation. Colours represent the average RGR at each experimental site. Species level survival after 1270 days planted at experimental locations is reported in Fig. 2 Each panel corresponds to data for each of the study species.

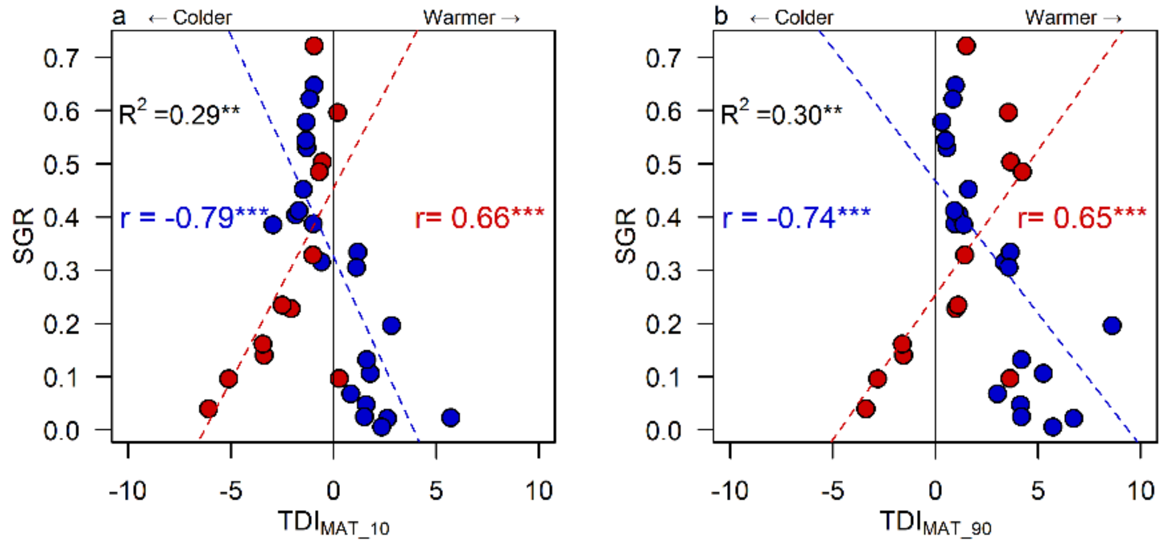

Supplementary Figure 3 | Relationship of scaled growth rate (SGR) and species thermal displacement from thermal optimum (TDI). TDI was estimated using: (a) MAT 10th percentile (TDI_{MAT_10}) and (b) MAT 90th percentile (TDI_{MAT_90}). A high absolute value of TDI_{MAT_10} (or TDI_{MAT_90}) indicates a high thermal displacement from species T_{opt}; this can be due to either a large difference between species T_{opt} and site MAT 10th percentile (or MAT 90th percentile), a low standard deviation of temperature across a species range, or both. A negative value of TDI_{MAT_10} (or TDI_{MAT_90}) means species T_{opt} is larger than site MAT10th percentile (or MAT 90th percentile), and a positive value indicates that species T_{opt} is lower than the site MAT10th percentile (or MAT 90th percentile). Data for montane and lowland species are shown in red and blue, respectively. R^2 values for the regression between SGR and TDI_{MAT_10} and between SGR and TDI_{MAT_90} and corresponding significance are shown and can be used to compare across panels and with Fig. 4. Pearson r and significance for each dashed line, which represent a linear model fit, correspond to montane and lowland species. Note that Pearson r is comparable within each panel but not across panels.

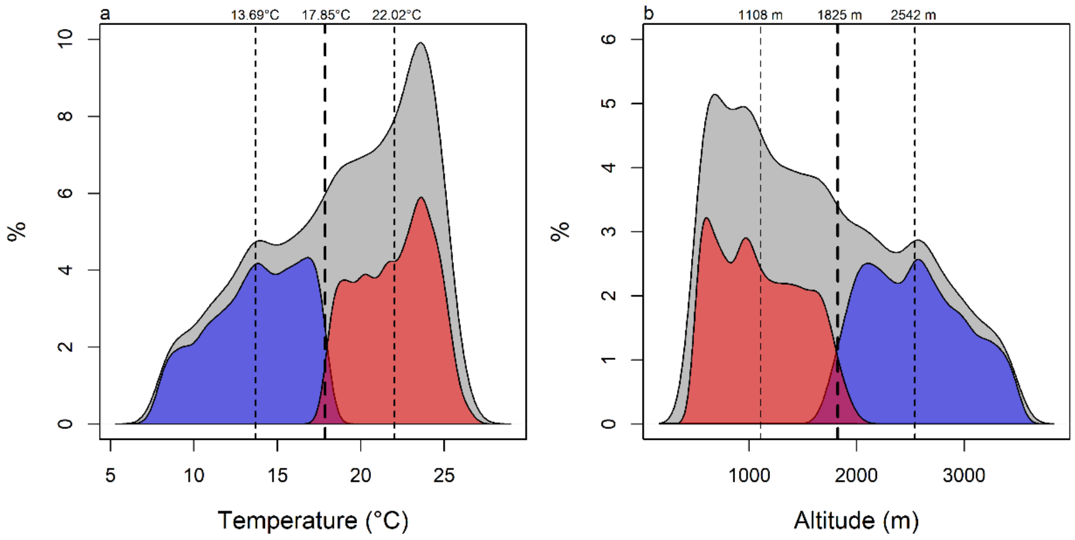

Supplementary Figure 4 | Density plot of temperature and elevation across the Colombian Andes. The grey area represents the whole dataset composed of 50,000 random data points of mean annual temperature (a) and altitude (b), blue and red areas represent thermal and altitudinal spaces of cold and warm montane areas respectively. Mean annual temperature (a) and mean altitude (b) are given for each group at the top of each panel (dashed lines). Bold dashed lines in both panels indicate the breaking point between the two groups. The y-axis represents the percentage of temperature or elevation data points from the whole data set (50,000 points).

Supplementary References

1. Wright, S. J. *et al.* Potassium, phosphorus, or nitrogen limit root allocation, tree growth, or litter production in a lowland tropical forest. *Ecology* **92**, 1616–1625 (2011).
2. Vadeboncoeur, M. A. Meta-analysis of fertilization experiments indicates multiple limiting nutrients in northeastern deciduous forests. *Can. J. For. Res.* **40**, 17666–1780 (2010).
3. van Do, T. *et al.* Monitoring fine root growth to identify optimal fertilization timing in a forest plantation: A case study in Northeast Vietnam. *PLoS One* **14**, 1–14 (2019).
4. Zhao, Q. & Zeng, D. H. Nitrogen addition effects on tree growth and soil properties mediated by soil phosphorus availability and tree species identity. *For. Ecol. Manage.* **449**, 117478 (2019).
5. Homeier, J. *et al.* Tropical Andean Forests Are Highly Susceptible to Nutrient Inputs-Rapid Effects of Experimental N and P Addition to an Ecuadorian Montane Forest. *PLoS One* **7**, (2012).
6. Vossen, P. Changing pH in Soil. *Univ. Calif. Coop. Ext.* **11**, 1–2 (2006).
7. Horneck, D. A., Sullivan, D. M., Owen, J. . & Hart, J. M. Soil Test Interpretation Guide | OSU Extension Catalog |. *Oregon State Univ.* **EC 1478**, 12 (2011).
8. Santiago, L. S. *et al.* Tropical tree seedling growth responses to nitrogen, phosphorus and potassium addition. *J. Ecol.* **100**, 309–316 (2012).
